# Effect of Hiwa syrup, a Persian Medicine Product, on Autism Symptoms and in Children with Autism Spectrum Disorders: A Randomized Double-Blinded Clinical Trial

**DOI:** 10.31661/gmj.v13i.3553

**Published:** 2024-10-09

**Authors:** Mohsen Dayani, Mehrdad Karimi, Seied Amirhosein Latifi, Mohammad Bagher Saberi Zafarghandi, Mehdi Salehi

**Affiliations:** ^1^ Department of Traditional Medicine, School of Medicine, Arak University of Medical Sciences, Arak, Iran; ^2^ Department of Iranian Medicine, Faculty of Iranian Medicine, Tehran University of Medical Sciences, Tehran, Iran; ^3^ Traditional and Complementary Medicine Research Center, Arak University of Medical Sciences, Arak, Iran; ^4^ School of Behavioral Sciences and Mental Health (Tehran Institute of Psychiatry), Iran University of Medical Sciences, Tehran, Iran; ^5^ Traditional and Complementary Medicine Research Center (TCMRC), Department of Traditional Medicine, School of Medicine, Arak University of Medical Sciences, Arak, Iran

**Keywords:** Autism Spectrum Disorder, Eye Contact, Herbal Medicine, Persian Medicine

## Abstract

Background: Autism spectrum disorder (ASD) is a pervasive neurodevelopmental condition characterized by challenges in communication, social interaction, sensory processing, emotional regulation, and repetitive behaviors. Eye contact, a crucial diagnostic and evaluative marker for autism, plays a significant role in enhancing social communication and educational abilities. Given the complexity of ASD and the absence of a definitive cure, attention has turned to traditional and complementary medicine for potential therapeutic options. Assess the efficacy of the Persian Medicine, Hiwa syrup, which consists of consisted of apple (Malus domestica Borkh.) fruit, quince (Cydonia Oblonga Mill.) fruit, basil (Ocimum basilicum L.), green cardamom (Elettaria cardamomum), and sandalwood (Santalum album Linn), in ameliorating autism symptoms and extending eye contact duration in children diagnosed with ASD.Materials and Methods: A double-blind randomized clinical trial involved 60 children (3-5 years) with level 1 autism, randomly assigned to intervention and control (placebo) groups. The intervention group received Hiwa syrup for eight weeks alongside routine therapy. Autism status was assessed using Persian version of Gilliam Autism Rating 2nd edition (GARS-2) questionnaire, and eye contact duration in response to auditory stimuli was measured pre and post-intervention. SPSS software version 25 (SPSS Inc., Chicago, IL, USA) was used for data analysis.Results: The Hiwa syrup group exhibited a significant decrease in the mean GARS-2 score from 75.03 ± 7.83 to 69.47 ± 5.87 (P=0.01) with a mean difference of 5.56 ± 3.12. This decrease surpassed that of the placebo group. Furthermore, the intervention group showed a significant increase in eye contact duration, from (7.90 ± 3.81 seconds to 9.26 ± 3.21 seconds (P=0.05), with a mean difference of 1.36±1.88 seconds. In contrast, the placebo group exhibited a smaller increase, from (7.50 ± 2.21seconds to 7.83 ± 2.91 seconds (P=0.64), with a mean difference of 0.33 ± 0.48 seconds.Conclusion: The polyherbal product from Persian Medicine appears to be effective in ameliorating autism symptoms and extending the duration of eye contact in children diagnosed with ASD. Further clinical trials are essential to validate the efficacy of this product in treating autism spectrum disorders.

## Introduction

Autism spectrum disorder (ASD) denotes a pervasive neurodevelopmental condition characterized by difficulties in communication and social interaction, sensory processing defects, inappropriate emotions, and repetitive and limited behaviors [1]. According to global statistics, its prevalence has increased in the last few decades [2]. Estimates from the ADDM Network for 2020 indicate an ASD prevalence rate of 27.6 per 1000 children aged 8 years, surpassing previous investigations [3]. Besides individual and family consequences, such as heightened anxiety and isolation among families with autistic children, autism imposes substantial economic costs on countries [2].

Impaired eye contact, a criterion indicating deficits in nonverbal social communication, is a hallmark feature of ASD and can be observed in both children and adults with ASD [1][4]. Scientifically and neurologically, eye gaze plays a significant role in ASD development, serving as an indicator for social interactions among individuals with autism [5]. Atypical eye contact can impact attention, memory, and various cognitive, emotional, and behavioral aspects of autistic individuals, significantly influencing social interactions from childhood through adulthood [5][6] Conversely, increasing eye contact may enhance learnability and improve certain skills [7].

While a definitive and permanent drug treatment for ASD has not been discovered, pharmacological interventions are used to alleviate comorbidities and associated symptoms, including aggression and irritability, often involving antidepressants, antipsychotics, anticonvulsants, and stimulants [8]. Monitoring the efficacy and adverse effects of pharmacologic interventions poses a therapeutic challenge due to the unique conditions of these patients [8][9]. Additionally, some evidence supports the effectiveness of cognitive, educational, and behavioral interventions, but the complexity of ASD’s pathophysiology necessitates more precise clinical studies [10].

Several studies have evaluated the efficacy of herbal medicine, supplements, and complementary medicine methods on ASD patients [11][12][13]. The use of complementary medicine is common among children and adults with ASD, likely driven by parents’ concerns about the effectiveness and safety of drugs. However, insufficient scientific evidence exists for the effectiveness and safety of complementary medicine interventions, and a knowledge gap persists among physicians regarding the use of complementary medicine in ASD [14]. Therefore, it is logical to conduct research with robust methodologies to evaluate the effects of complementary medicine in autistic patients

In Persian Medicine (PM), there is no exact term for autism; however, traditional medicine texts contain information about some children’s neurological and behavioral disorders [15]. Clinical trials have revealed the efficacy of on certain behavioral and neurological abnormalities, including improvements in Attention deficit hyperactivity disorder (ADHD), convulsions, and cognitive performance in children under the age of 18 [16][17][18]. Despite this, no clinical trials have been published on the effectiveness of PM on ASD. This study aims to explore the effectiveness of a Persian Medicine product (PMP), "Hiwa" syrup, which consists of consisted of apple (Malus domestica Borkh.) fruit, quince (Cydonia Oblonga Mill.) fruit, basil (Ocimum basilicum L.), green cardamom (Elettaria cardamomum), and sandalwood (Santalum album Linn). The syrup is intended to address the symptoms of autism and the duration of eye contact in response to auditory stimuli, a crucial criterion for assessing children with ASD. The selection of this drug for patients is based on the properties of its components from the perspective of Persian medicine, which improves brain and heart function, as well as the observed clinical experiences of the effectiveness of the drug and its components in some behavioral disorders, such as depression and anxiety.

## Materials and Methods

Ethical Consideration

This study received approval from the local ethics committee of Arak University of Medical Sciences, Arak, Iran (approval code: IR.ARAKMU.REC.1400.254) and adhered to the Declaration of Helsinki. All necessary considerations for conducting research on children with ASD were observed. Informed consents were obtained from parents after the researcher provided detailed explanations about the study. Since the study included children who did not require drug therapy and underwent regular occupational therapy sessions, there was no implication of treatment deprivation. The study was also registered at the Iranian Registry Center for Clinical Trials (registration code: IRCT20220628055306N1).

Material

The investigated PMP, Hiwa syrup, consisted of apple (Malus domestica Borkh.) fruit, quince (Cydonia Oblonga Mill.) fruit, basil (Ocimum basilicum L.), green cardamom (Elettaria cardamomum), and sandalwood (Santalum album Linn). Procured from a herbal medicine store in Tehran in 2022, the botanical authenticity of its components was confirmed by a botanist at the Herbarium of the Faculty of Pharmacy, Tehran University of Medical Sciences, Iran. The corresponding herbarium vouchers for sandalwood, basil, green cardamom, apple, and quince are PMP-946, PMP-3330, PMP-4612, PMP-4612, and PMP-4613, respectively, deposited at the Herbarium of Tehran University of Medical Sciences. PMP syrup was compared with a placebo syrup, prepared as a sugar solution in water for its sweet taste. Both formulations were dispensed in indistinguishable opaque bottles with identical visual attributes.

Study Design

This double-blinded randomized clinical trial took place at Golhay-e Behesht Autism Center in Qom (also spelled "Ghom), the capital of Qom province, located 125 kilometers south of Tehran on the boundary of the central desert of Iran, from December 2022 to February 2023. After confirming the autism diagnosis through an initial examination by a pediatric psychiatrist, parents underwent interviews with an experienced occupational therapist to determine the level of autistic subjects using the Gilliam Autism Rating 2nd edition (GARS-2) questionnaire [19]. Our research population was selected from children with ASD at the Golhay-e Behesht Autism Center in Qom, who had previously been evaluated using GARS-2 questionnaire by the center’s psychologist for primary screening. We therefore used the same questionnaire for pre-test and post-test assessments in this research. Only ASD subjects with level 1 were included. Subsequently, the duration of eye contact was evaluated and recorded by the occupational therapist. Subjects were then randomly assigned to intervention and control groups using block randomization. The intervention group received PMP, Hiwa syrup, for eight weeks at a dosage of 0.33 mg/kg (equivalent to 4-6 cc) three times a day. Simultaneously, the control group received a placebo syrup with an identical dosage. Parents of the children were provided with essential training on the accurate administration of the intervention according to the specified dosage. They were also encouraged to reach out to the researcher for any queries or issues during the study period. Both groups were assessed post-intervention by the same occupational therapist regarding eye contact duration and (GARS-2) questionnaire score.

Study Population

Participants were recruited via available sampling from autistic children aged 3-5 years seeking occupational therapy at Golhay-e Behesht Autism Center and not undergoing pharmacologic treatment. After primary evaluation and obtaining informed consent, eligible participants meeting inclusion criteria were enrolled.

Inclusion Criteria

(1) Definitive diagnosis of autism approved by a pediatric psychiatrist; (2) ASD with level 1 (mild characteristics of autism) according to The American Psychiatric Association’s Diagnostic and Statistical Manual of Mental Disorders (5th ed.) (DSM-5); (3) Aged between 3-5 years; (4) IQ higher than 70.

Exclusion Criteria

(1) Taking medication for ASD such as antipsychotics, anti-anxiety, and anti-convulsants; (2) Any adverse effects related to interventions; (3) Parents’ unwillingness to continue participating in the research project for any reason; (4) Self-injury or aggressive behaviors; (5) Severe hearing or visual impairments; (6) Afflicted with a neurological condition such as epilepsy or cerebral palsy, or a psychiatric ailment distinct from ASD.

Outcome Measures

The first outcome of this study was the assessment of status of ASD patients using validated and reliable Persian version of GARS-2 questionnaire [19]. The GARS-2, tailored for individuals aged 3-22 years, is a behavioral assessment tool aligned with DSM-IV criteria for Autism. Comprising three subscales (stereotyped behaviors, communication, and social interaction), each with 14 items, the GARS-2 encompasses a total of 42 items. Respondents are tasked with evaluating the frequency of examinee behaviors on a 4-point Likert scale, spanning from "Never Observed" to "Frequently Observed". The higher the score, the more severe the symptoms of autism. The questionnaires were completed by interviewing the child’s parents before and after the intervention [19][20].

The second outcome of this study was the duration of eye contact in response to auditory stimuli. Operationalized as the participant’s head and eye movement directed towards establishing direct eye contact with the experimenter’s eyes [21], the occupational therapist measured the duration of eye contact by calling the child during play. The duration was recorded using a stopwatch and documented in the Eye Contact Duration Chart. To mitigate bias, the same occupational therapist conducted both pre and post-intervention evaluations. This evaluation method is commonly employed in autism rehabilitation clinics to assess patient conditions.

Randomization and Blinding

Participants were randomly assigned to two groups using block randomization with a 1:1 allocation ratio, resulting in 30 patients per group. Prior to the trial’s commencement, a computer-generated randomization program, GraphPad (https://www.graphpad.com), was used to generate the random numbers. Blinding was effectively achieved as the containers for PMP and placebo syrups shared identical shape and size, along with a similar appearance in their syrups. Consequently, patients were unaware of their drug allocations. Furthermore, the occupational therapist, researchers, and statisticians involved in the study were all blinded to the allocation of subjects.

Safety Assessment

All components of the product adhered to the allowed therapeutic dose according to the Physician’s Desks Reference (PDR) for Herbal Medicine. Specifically, Elettaria cardamomum dose in syrup was under 1 gram, and Santalum album Linn dose in syrup was under 10 grams daily [22]. Furthermore, the dosage of all components of the Hiwa syrup was in accordance with the results of conducted clinical trials, animal or in vitro models [23][24][25][26][27]. The researcher consistently monitored patients for any potential side effects, and contact information was provided to parents for immediate communication in case of concerns.

Sample Size

The required sample size in each group was calculated using the formula, considering a significance level of 5% and a study power of 80%. With a 10% attrition rate, a minimum of 30 individuals in each group and a total of 60 samples were determined for this study.

**Figure:**
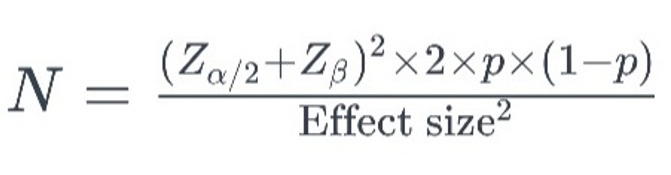


Statistical Analysis

Data analysis employed t-test, chi-square, and Fisher’s exact test. Additionally, non-parametric Mann-Whitney and Wilcoxon tests were used to compare variable values of eye contact duration before and after the intervention. SPSS version 25.0 (SPSS Inc., Chicago, IL, USA) was utilized for data analysis.

## Results

**Figure-1:**
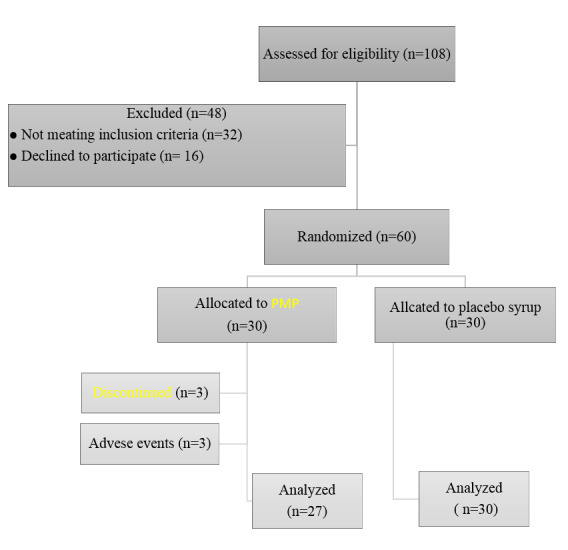
Figure 1.The flowchart of the trial

In the initial screening of 108 autistic children, 76 were deemed eligible for inclusion in the study. The intervention and control groups each comprised 30 subjects, totaling 60 participants. At the conclusion of the study, 27 subjects in the intervention group and all 30 subjects in the control group completed the study (Figure-1). The majority of study subjects were boys, with a predominant age of 4 years, and the mothers of most participants had at least a diploma-level education or higher (Table-1). Chi-square tests revealed no significant differences between the two groups regarding gender, age, or the education level of mothers. After the intervention, the improvement of autism symptoms was observed in the patients of the intervention group, which was consistent with the significant change of the GARS-2 questionnaire. While the placebo group had no significant change in the results (Table-2). Initially, the duration of eye contact did not significantly differ between the two groups. The study results indicated that the change in the control group was not statistically significant. However, in the intervention group, there was a significant increase in eye contact duration from 7.90±3.81 to 9.26±3.21 (P-value=0.05). Moreover, the comparison between the two groups demonstrated the effectiveness of PMP compared to placebo in significantly enhancing eye contact duration (P<0.001, Table-3). PMP demonstrated good tolerability for the majority of subjects in this study. Nonetheless, three children were excluded due to increased restlessness. The follow-up of these patients revealed that symptoms attributed to the drug were resolved upon discontinuation.

## Discussion

**Table:** Table1. Charectristics of the Subjects of the Trial

**Item**	**PMP group (n=30) **	**Placebo group** **(n=30)**	**P-value**
**Gender (n, %)**			
**Boy**	17 (56.7)	19 (63.3)	0.59
**Girl**	13 (43.3)	11 (36.7)	
**Age range (n, %) **			
**3 - 3.5 y**	5 (16.7)	5 (16.7)	
**3.5- 4 y**	14 (46.7)	11 (36.7)	0.69
**4- 5 y**	11 (36.7)	14 (46.7)	
**Mother's educational level (n, %) **			
**Under diploma**	4 (13.3)	3 (10)	
**Diploma**	16 (53.3)	14 (46.7)	0.84
**Bachelor's degree**	9 (30)	11 (36.7)	
**Master's degree and higher **	1 (3.3)	2 (2.6)	

**Table:** Table2. Comarision of Total Score of GARS-2 Questionare before and after Intervention

**Group**	**Total score of GARS-2 questionnaire **			**P-value**
	Before intervention (Mean ± SD)	After intervention (Mean ± SD)	difference (Mean ± SD)	
**PMP**	75.03 ± 7.83	69.47 ± 5.87	5.56 ± 3.12	0.01
**Placebo**	74.37± 6.96	73.63± 6.72	0.73 ± 7.83	0.73
**Between**				<0.001

In this randomized, double-blind clinical trial, the efficacy of Hiwa syrup, a PMP, was evaluated for treating symptoms of children with ASD. The results showed that Hiwa syrup was effective in reducing autistic symptoms and improving eye contact duration in children with ASD.

Given the absence of a definitive treatment for autism, the intricate pathophysiology of the disorder, and the recognized influence of environmental factors, researchers have turned their attention to exploring the effects of nutrition, diets, and supplements on the symptoms of autistic patients. Previous studies have delved into the efficacy of medicinal plants in addressing autism-related challenges. For instance, Chan et al. (2018) conducted a clinical trial examining the effects of a 6-month administration of intranasal herbal medicine on children with ASD, observing improvements in executive functions, prefrontal and anterior cingulate cortices activation, and enhancements in daily executive behaviors [28]. In a different study by Elangovan et al. (2023), an open-label investigation showcased the effectiveness of a natural formulation derived from Siddha plus oleation therapy on autistic children. Over a 90-day follow-up, improvements were noted in social communication, emotional responsiveness, speech, behavioral and cognitive aspects, as well as sensory issues [29].

Our study contributes to this body of research by demonstrating the positive effects of a polyherbal formulation derived from Persian Medicine, administered over eight weeks, in improvement of behaviors, social interaction, communication skills and enhancing the duration of eye contact in children with ASD. Described as beneficial for the brain and nervous system in Persian Medicine resources, the components of this product exhibit properties such as joyfulness, relaxation, calming, anti-anxiety, anti-depressant, and brain-strengthening [30][31].

According to Persian Medicine, the temperament of an individual is linked to the functioning of the brain. A body and brain's coldness may contribute to the development of mental and psychological impairments [32][33]. Recent research has demonstrated a connection between a cold temperament and certain mental disorders like depression, hopelessness and sleep disturbances [34][35][36][37][38]. Furthermore, a cross-sectional study indicated that children with ASD frequently display a cold and dry temperament. Considering that the components of our product embody warm temperament properties, they have a potential beneficial impact on the treatment of autism from the perspective of PM [39].

While specific research on the effects of the medicinal product's components on autism was not identified, our study documents their beneficial effects on the brain and nervous system. Experimental studies highlight the neuroprotective properties of apple and its derivatives, showcasing benefits for memory impairment and cognitive function. Additionally, apple has demonstrated the reduction of neuronal damage induced by oxidative stress in animal models, with positive effects on Alzheimer's disease and comparable antidepressant efficacy to imipramine [40][41][42]. Quince, another component, has shown promising impacts on neuropsychiatric disorders, enhancing hippocampal neurogenesis and improving physiological and behavioral markers in a rat model of depression [43]. Extracts from quince leaf have demonstrated effects on locomotor activity and anxiety-like behavioral changes in an animal model with schizophrenia [44]. Green cardamom has exhibited a reduction in oxidative stress and neuroinflammation in rat models, and perinatal exposure has led to memory and learning improvement in mice [45][46]. Basil, with documented neuroprotective, antianxiety, sedative, and antidepressant effects, has been associated with improved brain functions after cerebral injury in mice [47][48][49]. Sandalwood, another component, possesses neuroprotective properties, as evidenced in clinical studies on autism patients [50][51]. Given the high prevalence of comorbid psychiatric disorders such as depression in autistic patients [52], it can be hypothesized that the effect of Hiwa syrup on improving autism may be related to the improvement of affective symptoms.

The natural agents in our study's medicinal formula have antioxidant properties, a crucial aspect considering oxidative stress as a significant mechanism in the pathogenesis of autism. Oxidative stress contributes to toxicity and neuronal destruction, and studies support the benefits of antioxidants in controlling autism symptoms, including behavioral challenges, irritability, and hyperactivity [53]. Furthermore, inflammation plays a pivotal role in autism pathogenesis [54], and the anti-inflammatory effects of the components in PMP could be considered as one of the mechanisms of action. In addition, the bioactive compounds present in Hiwa syrup, including flavonoids and phenolic compounds, have been shown to exhibit anti-inflammatory, antioxidant, and neuroprotective properties, which may contribute to improved cognitive function [55][56]. The herbal formula also includes vitamins A, C, and E, which have been associated with ASD development. Thus, the presence of these vitamins may elucidate the mechanism of action of our study's polyherbal formula [57][58]. However, it is crucial to acknowledge the need for more precise clinical trials to validate the efficacy of PMP in treating ASD.

To date, this study stands as the inaugural randomized placebo-controlled clinical trial investigating the effectiveness of a naturally derived drug rooted in Persian Medicine for children with ASD. The robust methodology, incorporating a placebo as the control group, distinguishes our study and contributes to mitigating research bias, ultimately leading to more accurate conclusions. This approach aligns with a more rigorous standard compared to many similar published studies [11]. Despite these strengths, the study has limitations, including the notable weakness of not utilizing automatic eye contact measurement devices, such as eye trackers. Future research endeavors should consider addressing these limitations to further enhance the validity and comprehensiveness of investigations into the efficacy of interventions for autism spectrum disorders.

## Conclusion

**Table:** Table3. Duration of Eye Contact in Response to Auditory Stimuli in two Groups

	**Duration of eye contact (second) **			**P-value**
**Groups**	PMP group (n=27) Mean ± standard deviation	Placebo group (n=30) Mean ± standard deviation	difference (Mean ± SD)	
**Before intervention **	7.90±3.81	7.50±2.21	1.36±1.88	0.05
**After intervention**	9.26±3.21	7.83±2.91	0.33±0.48	0.64
**Between groups **				<0.001

The findings indicate that Hiwa syrup, a Persian Medicine product, is effective in improving symptoms associated with autism and the duration of eye contact in response to auditory stimuli among children with ASD. Given the significant role of eye contact in enhancing social interaction and improving the quality of life for these individuals, this product may offer beneficial effects for autistic patients. Nevertheless, further, more precise clinical trials are essential to substantiate and confirm the effectiveness of Hiwa syrup in the context of ASD.

## Acknowledgments

This paper has been published with the financial support of Deputy of Research and Technology at Arak University of Medical Sciences, Arak, Iran.

## Conflict of Interest

The authors affirm that there is no conflict of interest. The authors are solely accountable for the accuracy and integrity of the content within the paper.
